# Tumor probability mapping of fluorescence intensity during image-guided surgery of head and neck cancer

**DOI:** 10.1038/s41598-026-56078-4

**Published:** 2026-06-05

**Authors:** Akhilesh M Wodeyar, Sherin James, Benjamin B. Kasten, Nicolaus D. Knight, Carlos A. Gallegos, Ameer Mansur, Julian Barnhill, Anthony B. Morlandt, Harishanker Jeyarajan, Carissa M. Thomas, Bharat Panuganti, Anna G. Sorace, Eben L. Rosenthal, Jason M. Warram

**Affiliations:** 1 Department of Otolaryngology, University of Alabama at Birmingham, Volker Hall G082, 1670 University Boulevard, AL 35294 Birmingham, USA; 2 Department of Biomedical Engineering, University of Alabama at Birmingham, AL Birmingham, USA; 3 University of Alabama, Tuscaloosa, AL USA; 4 Department of Oral and Maxillofacial Surgery, Section of Oral Oncology, University of Alabama at Birmingham, AL Birmingham, USA; 5 Department of Radiology, University of Alabama at Birmingham, AL Birmingham, USA; 6 O’Neal Comprehensive Cancer Center, University of Alabama at Birmingham, AL Birmingham, USA; 7 Department of Otolaryngology-Head and Neck Surgery, Vanderbilt University, Nashville, TN USA

**Keywords:** fluorescence-guided surgery, tumor probability mapping, surgical margins, panitumumab-IRDye800, intraoperative imaging, predictive analytics, Cancer, Medical research, Oncology

## Abstract

Achieving negative surgical margins remains a critical determinant of local recurrence and survival in head and neck cancer (HNC) surgery. Current intraoperative margin assessment techniques, including frozen section analysis, suffer from sampling errors and procedural delays. Tumor-targeted fluorescence imaging offers real-time tumor visualization but lacks standardized quantitative approaches for clinical decision-making. We developed a Tumor Probability Mapping (TPM) framework using panitumumab-IRDye800 fluorescence imaging in 16 HNC patients. Ex vivo specimens and gross tissue sections were imaged using near-infrared fluorescence systems. A total of 5,442 regions of interest (ROIs) were manually distributed across fluorescence images of gross specimen sections validated by histopathology. Signal-to-background ratios (SBR) were calculated and used to train the following predictive models: generalized linear model fit standard logistic regression (MATLAB, glmfit), standard logistic regression (R, LOG), mixed-effects logistic regression (GLMER), and Bayesian mixed-effects regression (BRMS). Model performance was evaluated using receiver operating characteristic and area under the curve (ROC-AUC) analysis, sensitivity, specificity, along with beta-calibration and model fit. All models demonstrated excellent (> 90%) discriminative ability between tumor and normal tissue. The glmfit model, selected for clinical implementation, achieved 95.8% accuracy, 90.8% sensitivity, 98.8% specificity, and an AUC of 0.989 on test data. The final TPM algorithm provides real-time probability assessment of tumor presence based on fluorescence intensity quantified by histopathology validated historical data. TPM represents a significant advancement in fluorescence-guided surgery by converting qualitative fluorescence signals into quantitative probability assessments validated against histopathology. This approach provides surgeons with standardized, real-time tumor probability information that extends beyond qualitative assessments and/or binary threshold determinations, potentially improving surgical outcomes by enhancing margin assessment and reducing local recurrence rates.

**Supplementary Information:**

The online version contains supplementary material available at https://doi.org/10.1038/s41598-026-56078-4.

## Introduction

The standard of surgical care in head and neck cancer (HNC) aims for complete en bloc tumor resection with negative margins along with removal of residual microscopic tumor tissue^1,2^. Achieving R0 resections in HNC surgery can be challenging due to the complex anatomy within head and neck regions. A modify-as-you-go strategy without real-time guidance, which is common practice, can lead to irregular resection margins, particularly on the deep surface of tumors^3^. Extensive literature evidence suggests positive margins as a significant factor for local recurrence rate (LRR) and disease-free survival (DFS) for patients undergoing surgery for HNC^2,4–11^. Deep margin status has been shown to be a key prognostic factor, and tumors exhibiting deep invasion were shown to be associated with significantly higher rates of local and regional recurrence^12^. Inadequate margins have demonstrated prolonged operative time, challenges in orienting resected tissue for pathology, and reconstructive compromise^2^. Although achieving negative margins remains a significant clinical challenge during HNC surgery, surgical resection with negative margins is the only prognostic factor that can be controlled by the surgeon^8^. Frozen section analysis (FSA), first described over 75 years ago, is a well-established method for intraoperative margin assessment (IMA)^13–15^ but is limited by sampling errors and procedural delays^16^. Newer IMA approaches such as touch-print cytology, topical staining, optical techniques, and fluorescence imaging (FI) have since emerged as alternatives.

FI has evolved significantly since its early use in cancer detection, offering real-time, high-resolution visualization of tumors during surgery. Combined with the adavantage of spatial resolution and advances in tumor-targeted fluorescence (TTF) probes, FI has enabled precise localization of malignant tissues, improving surgical accuracy and oncologic outcomes^13,14^. TTF imaging employs fluorescent tracers designed to selectively bind to receptors or molecules expressed on malignant cells^15,16^. This technique serves as a bridge between conventional histopathological evaluation of resected specimens and optical imaging methods to enable intraoperative cancer confirmation. Due to its ability to provide immediate molecular-level tumor identification during surgical procedures, TTF is often characterized as molecular pathology. Recent analysis of the diagnostic accuracy of various emerging IMA technologies concluded that TTF imaging has excellent practical applicability and sensitivity relative to FSA^17^. Studies done over the last two decades in training and testing TTF technology, including clinical trials, have shown that TTF has excellent sensitivity in margin assessment and enables identification of small, otherwise undetectable satellite tumor tissues^16–21^.

Despite their many advantages, FI techniques still face several limitations that hinder their widespread clinical adoption^22^. The inherent qualitative nature of signal interpretation introduces variability and potential subjectivity while using FI in real time. Challenges such as tissue heterogeneity and autofluorescence impede accurate fluorescence assessment in routine clinical use^23^. Additionally, the lack of robust, pathology-validated fluorescence data complicates the translation of imaging findings into actionable surgical guidance. While current imaging technologies offer high-resolution, quantitative fluorescence capabilities, there is no model to accurately translate this information for real-time intraoperative guidance. These challenges highlight a significant gap in utilizing fluorescence data to establish a standardized, quantitative approach for tumor delineation.

In this report, we have conceptualized ‘tumor probability mapping’ (TPM), which correlates histopathology with fluorescence intensities normalized to background, referred to as the signal-to-background ratio (SBR). We employed multiple predictive models that were trained and tested on data derived from the SBR of 5,442 individual regions of interest (ROIs) manually delineated on fluorescent images of tissue sections from 16 HNC patients who underwent fluorescence-guided surgery (FGS) for HNC. These models can predict with high accuracy the percentage probability of a new SBR containing tumor during the surgery. This approach was designed in consideration of the established guidelines for FDA-directed human testing and approval of investigational optical imaging devices and contrast agents, which provide surgeons with information that extends beyond the visible light spectrum, thereby enhancing visual detection of tumor tissue^19^. By utilizing a color-coded lookup table validated with histopathology, a predictive tumor map can be overlaid onto the surgical field. This critical information aids surgeons while making surgical decisions as an alternative to relying on cutoffs or thresholds, which could hinder surgical decision-making and limit adherence to FDA guidelines. Here, we evaluate a TPM approach that is suitable for the clinical application to aid surgeons in real-time during FGS of HNC.

## Methods

### Ex vivo specimen and gross section fluorescence imaging

In accordance with the trial design (ClinicalTrials.gov identifier: NCT04511078), patients were administered a fixed 50 mg dose of panitumumab-IRDye800 intravenously 1–2 days before their scheduled surgery. Informed consent was obtained from all participants, and patient data were anonymized following institutional and federal ethical guidelines. The study procedures involving human specimens received approval from the Institutional Review Board at the University of Alabama at Birmingham.

Intraoperative fluorescence imaging was conducted in the operating room using an FDA-approved, open-field imaging system (LUNA, Novadaq, Ontario, Canada). Post-resection, the en bloc primary tumor specimens and gross fresh tissue sections (approximately 5 mm thick) were imaged using a closed-field near-infrared fluorescence (NIRF) imaging system (Pearl Impulse, LI-COR Biosciences, Lincoln, NE; excitation: 785 nm, emission: 820 nm). Ex vivo specimens were imaged immediately at the back table, and gross sections were imaged within one hour of tissue resection, following gross examination and sectioning by a pathologist in the surgical pathology laboratory.

Tissue samples were then fixed in 10% neutral buffered formalin, paraffin-embedded, and processed for histopathological evaluation according to institutional standard operating procedures. NIRF images of fresh gross tissue sections were analyzed using Image Studio software (version 5.2.5, LI-COR Biosciences). Samples from 16 consented patients (both male and female) enrolled in the phase II clinical trial were retrieved from the UAB Pathology Core for downstream pathological analysis. Hematoxylin and eosin (H&E) staining was used to assess tumor morphology and normal tissue architecture, including stromal features.

### Correlation of histology with intraoperative imaging

**Fig. 1:**
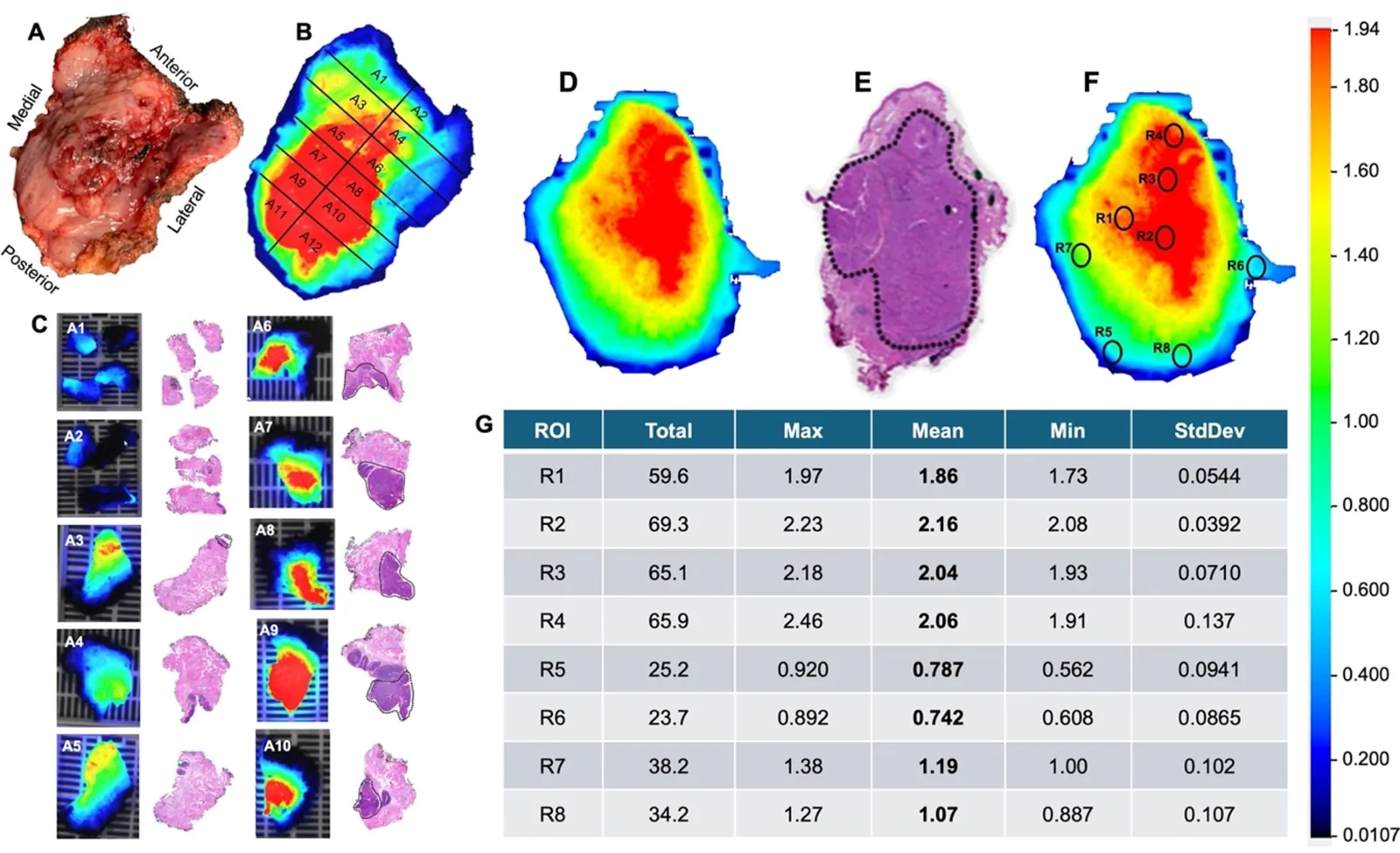
Workflow for ex vivo fluorescence imaging and histopathological correlation in head and neck cancer specimens. **(A)** Bright-field image of fresh buccal mucosa specimen with surgical margins annotated (anterior, posterior, medial and lateral). **(B)** Ex vivo fluorescence image of the same whole specimen showing grossing slice locations (black lines) as designated by the pathologist for subsequent sectioning. **(C)** Individual tissue slices processed for both closed-field fluorescence imaging (left row) and corresponding hematoxylin and eosin (H&E) histological sections (right row), demonstrating spatial correlation between fluorescence signal and tissue architecture. **(D & E)** Workflow illustrating the image alignment process between ex vivo fluorescence images and histological sections with pathologist-annotated tumor margins (black dotted line) delineating malignant tissue from adjacent normal tissue. **(F)** Regions of interest (ROI) selection on registered fluorescence image using Image studio 5.2 software, with circular ROIs placed on tumor tissue (black circles) and normal tissue for quantitative analysis. **(G)** Quantitative fluorescence analysis showing mean fluorescence intensity (MFI) values extracted from each ROI, which were subsequently used to calculate signal-to-background ratio (SBR) for tumor-to-normal tissue contrast assessment. Fluorescence scale (MFI); all the imaging was done on Pearl (closed-field fluorescence imaging system). H&E, hematoxylin and eosin; MFI, mean fluorescence intensity; ROI, region of interest; SBR, signal-to-background ratio.

The complete dataset is comprised of 5,442 ROIs from 16 patients for analysis, which included 2,361 tumor ROIs and 3,081 normal tissue ROIs. These were partitioned into separate patient sets for training (10 consecutive patients, ~ 70% ROIs) and testing (6 consecutive patients, ~ 30% ROIs). Pathological status (tumor vs. normal) was assigned to each ROI based on H&E evaluation by a trained H&N pathologist. If any component of the ROI was tumor, it was classified tumor. To spatially correlate intraoperative fluorescence with histologically verified tumor margins, a comprehensive ex vivo tumor diagram was constructed for each patient (Fig. 1). These maps integrated intraoperative gross fluorescence images and H&E-verified pathology sections. Gross section orientation was maintained throughout tissue processing to enable alignment between fluorescence imaging, pathology sections, and tumor annotation. Tumor boundaries were marked on H&E slides by a board-certified pathologist using a fine-tipped marker to demarcate the interface between tumor and normal tissue. A total of 250 slides, out of which 102 FFPE tumor sections were diagnosed as squamous cell carcinoma (SCC) and 148 sections without tumor across 16 patients, were included for analysis. Fluorescence images of the corresponding gross fresh tissue sections were then spatially matched to compare annotated boundaries to assess the accuracy of panitumumab-IRDye800 distribution in reflecting tumor localization. ImageStudio 5.2 software was used to draw fixed-size ROIs and quantify mean fluorescence intensity (MFI) within each ROI across each fluorescence image of the bread-loafed specimens, which were compared to the corresponding pathologic section to ensure gold-standard diagnostic concordance (Fig. 1). Tissue sections without tumor served as background references for each patient, from which a minimum of 10 ROIs were drawn and averaged to calculate the background fluorescence signal. Signal-to-background ratios (SBRs) were computed by dividing tissue MFI (total fluorescence/pixel area) values by matched background MFI values per patient. To account for tissue heterogeneity and minimize variability due to autofluorescence, all fluorescence data acquisition, exposure settings, and ROI quantifications were standardized.

### Tumor probability prediction models

To derive fluorescence-based predictive metrics of tumor presence, logistic regression analysis was applied across all ROIs. The TPM pipeline and image processing code were developed in MATLAB 2023b (MathWorks, Natick, MA), using glmfit as an inline logistic regression function for pixel-wise application. The mixed-effects (GLMER) and Bayesian mixed-effects (BRMS) models were performed using R studio (version 4.4.1) (lme4 and brms, respectively). To further validate logistic regression results from MATLAB, standard logistic regression in R (LOG) was performed to confirm implementation consistency across software environments. These models estimated the probability of a given pixel or region representing tumor tissue based on its MFI or SBR value. To quantify uncertainty while accounting for clustering of ROIs within patients, 95% confidence intervals for all performance metrics were estimated using a patient-level bootstrap with 2,000 resamples of the independent test cohort.

### TPM image generation

The calibrated SBR-based logistic regression model was applied pixel-wise to generate TPM images overlaid on surgical images. Surgical fluorescence and brightfield imaging data from selected patients across the training and test cohorts were exported from the PEARL (closed-field system for ex-vivo specimen imaging) and LUNA (open-field system for in-vivo real-time imaging) imaging systems as TIFF and DICOM files, respectively. SBR-based logistic regression predictions generated pixel-wise tumor probability heatmaps with color lookup tables using custom scripts (available upon request) in MATLAB to produce TPM images and videos. To convert the DICOM images into SBR format, video signal intensity was scaled on a patient-wise basis using the highest histologically confirmed tumor SBR from the respective histological sectioning. Upon conversion, the logistic regression equation was applied pixel-wise to determine tumor probability at each frame. Final TPM image sequences were overlayed on the original image sequences and exported as a video for visualization.

### Statistical analysis

Statistical analysis was performed using R Studio. Receiver operating characteristic and area under the curve (ROC-AUC) analysis was performed to evaluate the diagnostic performance of fluorescence intensity in discriminating tumor from normal tissue. Sensitivity, specificity, and area under the curve (AUC) were calculated to determine the accuracy of fluorescence-based tumor detection representing the probability of correctly distinguishing tumor from normal tissue in both the training and test set. Paired comparisons of MFI and SBR were performed using the Wilcoxon rank-sum test. AUC-ROC comparisons were conducted using DeLong’s test. *P* < 0.05 was considered significant for statistical analysis.

## Results

### Tumor probability prediction model performance (training set)

**Fig. 2:**
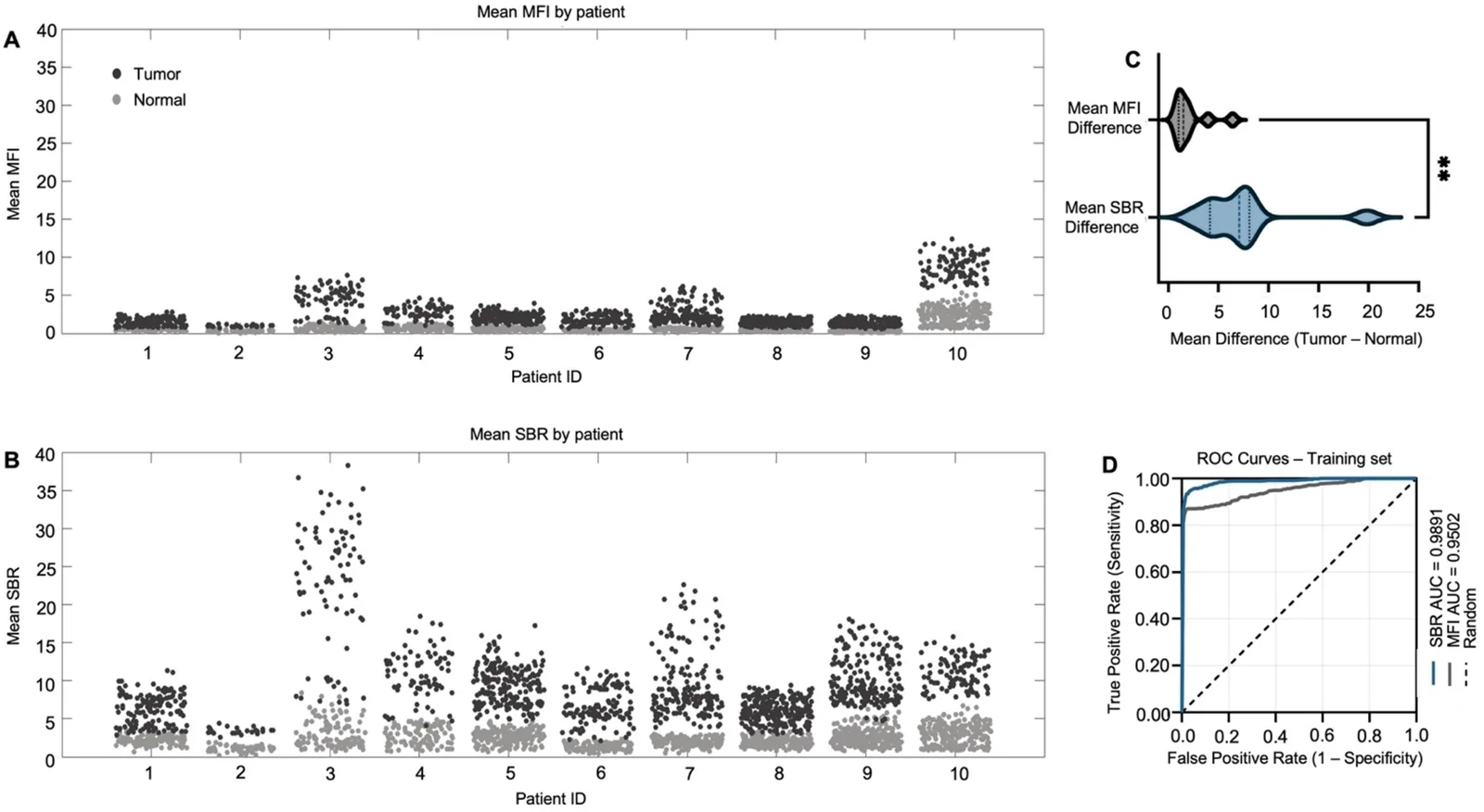
Comparative analysis of MFI and SBR for tumor detection in head and neck cancer specimens. Quantitative assessment of fluorescence-based tissue characterization metrics in a testing cohort of head and neck cancer patients (*n* = 10). **(A)** Dot plot displaying MFI values for individual ROI from tumor and normal tissue across the first 10 patients in the testing set. Each dot represents a single ROI measurement, with patient identification on the x-axis and MFI values on the y-axis. **(B)** Corresponding dot plot showing SBR distribution for the same patient cohort and ROI locations. **(C)** Direct comparison of MFI versus SBR data points using violin plot, **p < 0.001 (Wilcoxon signed-rank test). **(D)** ROC curve analysis comparing the diagnostic performance of MFI and SBR metrics for tumor detection. AUC, area under the curve; MFI, mean fluorescence intensity; ROC, receiver operating characteristic; ROI, region of interest; SBR, signal-to-background ratio.

We first evaluated a logistic regression model in MATLAB using the ‘glmfit’ function for the training set (comprised of the first 10 patients) with the mean signal-to-background ratio (MeanSBR) and MFI as ground truth predictors (Fig. 2). MeanSBR better differentiated the classes and outperformed MFI in all metrics (Fig. 2C). Paired differences between tumor and normal tissue ROI values were significantly greater for SBR compared to MFI metrics (*p* < 0.001). The enhanced separation between tumor and normal tissue measurements demonstrates improved discriminatory power of SBR compared to raw MFI values. SBR demonstrates superior diagnostic accuracy with an area under the curve (AUC) of 0.984 compared to MFI (AUC = 0.950), confirming SBR as the more robust metric for fluorescence-based tissue characterization (Fig. 2D). 10-fold cross-validation across the training set of 10 patients demonstrated consistent F1 scores exceeding of 0.935 for all models, indicating excellent balance between precision and recall.

### Tumor probability prediction model performance (test set)

**Fig. 3:**
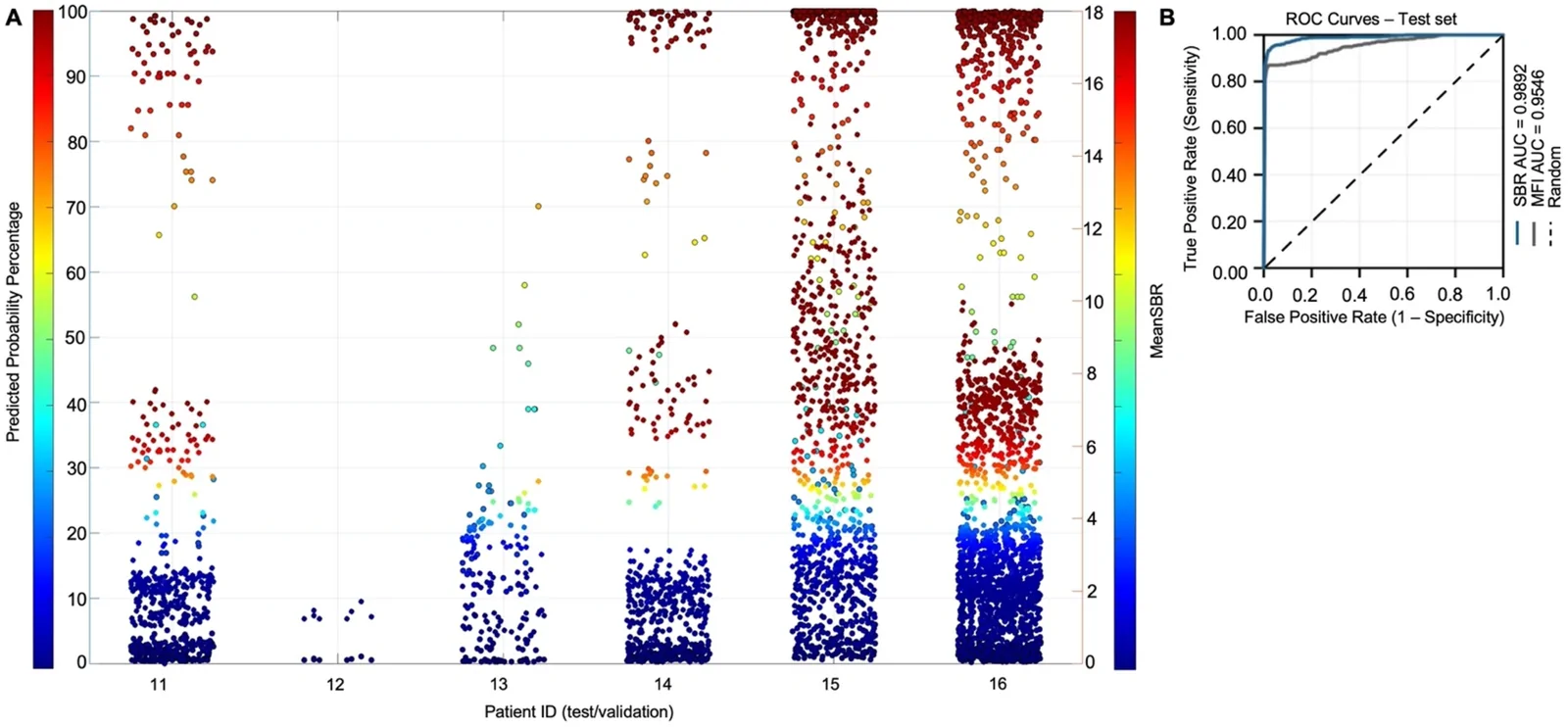
SBR-based tumor detection model in an independent (test set) patient cohort. Independent analysis of fluorescence-based tissue characterization in the test cohort of head and neck cancer patients (*n* = 6). **(A)** Dot plot with color mapping displaying the percentage probability of tumor detection for individual ROIs across the test set of 6 patients. Each point represents a single ROI measurement, with corresponding color scale to the predicted probability of malignancy based on the linear regression model using SBR values. **(B)** ROC curve analysis comparing MFI and SBR performance in the test cohort.

Following model development using the training set, the trained logistic regression model was applied to the test cohort of 6 additional patients to evaluate generalizability and predictive accuracy (Fig. 3A). The linear regression model relates tumor probability to mean SBR values (Probability vs. MeanSBR), demonstrating the predictive capability of SBR-based tissue classification in previously unseen patient data. Just like the training set, SBR maintains superior diagnostic accuracy with AUC = 0.989 compared to MFI (AUC = 0.955), confirming the robustness and generalizability of SBR as the preferred metric for fluorescence-based tumor detection across independent patient populations (Fig. 3B).

The confusion matrix for the test set revealed 1,423 true negatives, 888 true positives, 90 false negatives, and 18 false positives. False negative and false positive classifications of ROIs in the test set corresponded to intermediate SBR values with varying calculated probability for malignancy (Supplementary Table 1). This generalized linear model fit (glmfit) logistic regression model served as a simplified benchmark and demonstrated strong classification performance. Although it does not account for intra-patient correlation, its high AUC and balanced metrics support the feasibility of using fluorescence MeanSBR as a predictor of tumor presence. We further developed and compared two additional statistical models: mixed-effects logistic regression (GLMER), and Bayesian mixed-effects logistic regression (BRMS). All models utilized the MeanSBR derived from fluorescence imaging as the primary predictor variable, as the previous glmfit model did. Each model was trained on the same dataset partition to ensure fair comparison, with particular attention given to addressing potential intra-patient correlation in the mixed-effects models by including random intercepts for each patient. The primary purpose of the multi-model comparison was to evaluate the added value of accounting for inter-patient correlation (GLMER) and uncertainty quantification (BRMS) relative to the standard logistic baseline. R based standard logistic regression (LOG), which is mathematically equivalent to standard logistic regression (MATLAB, glmfit), was used to ensure consistent results across software vendors. Results of this software-validation analysis is shown in supplemental Table 4.

SBR-based logistic regression performance of the full dataset of 5,442 ROIs across training and testing patient cohorts is shown in Supplementary Fig. 1. This detailed visualization enabled assessment of model performance across the full spectrum of SBR values, identification of potential optimal thresholds, and evaluation of prediction accuracy patterns across all 16 patients.

### Classification performance

All three models demonstrated excellent discriminative ability between tumor and normal tissue based on MeanSBR values. When applied to the separate datasets, all models maintained high accuracy (Tables 1 and 2). The glmfit model achieved an area under the ROC curve (AUC) of 0.984 for the training dataset (Table 1) and an overall accuracy of 95.5% with a sensitivity of 90.8% and specificity of 98.8% for the testing dataset (Table 2). Confidence intervals for all performance metrics were derived using a patient-level bootsrap, where entire patients, rather than individual ROIs, were resampled. Despite the large number of ROIs, the resulting confidence intervals remained relatively wide and overlapping across models, indicating that variability in fluorescence signal characteristics between patients rather than the sheer number of ROIs was the dominant source of uncertainty. Similar performance metrics between training and test datasets suggest that TPM is robust to variations in ROI count, which supports generalizability of this approach.

**Table 1:** Performance metrics (95% CI) on training dataset @ 0.5 threshold:

Model	Accuracy	Sensitivity (Recall)	Specificity	PPV (Precision)	NPV	AUC	F1 Score
Standard Logistic regression (MATLAB)	92.9% (88.9–95.7)	90.5% (82.3–96.7)	94.9% (90.7–98.8)	93.8% (87.1–98.6)	92.2% (85.8–97.4)	0.984 (0.969–0.994)	0.921 (0.871–0.954)
Mixed-effects logistic regression	92.9% (89.4–95.8)	90.6% (83.0–97.3)	94.8% (90.6–98.7)	93.6% (87.4–98.5)	92.3% (85.9–97.8)	0.984 (0.971–0.994)	0.921 (0.876–0.956)
Bayesian mixed-effects logistic regression	92.9% (89.4–95.8)	90.5% (82.9–97.2)	94.9% (90.9–98.8)	93.8% (87.7–98.6)	92.2% (85.8–97.8)	0.984 (0.971–0.994)	0.921 (0.877–0.956)

Brier scores (95% CI): Standard logistic regression (MATLAB): 0.0504 (0.0318–0.0767), Mixed-effects logistic regression: 0.0619 (0.0355–0.0932), Bayesian mixed-effects logistic regression: 0.0504 (0.0315–0.0735).

**Table 2:** Performance metrics (95% CI) on test dataset *@ 0.5 threshold*.

Model	Accuracy	Sensitivity (Recall)	Specificity	PPV (Precision)	NPV	AUC	F1 Score
Standard Logistic regression (MATLAB)	95.5% (84.2–98.2)	90.8% (54.6–98.2)	98.8% (97.5–100)	98.0% (97.6–100)	94.1% (79.4–98.4)	0.989 (0.965–0.999)	0.943 (0.707–0.980)
Mixed-effects logistic regression	95.7% (85.0–98.2)	91.3% (58.4–98.4)	98.6% (97.3–100)	97.8% (97.4–100)	94.4% (80.0–98.6)	0.989 (0.966–0.999)	0.944 (0.738–0.980)
Bayesian mixed-effects logistic regression	95.5% (84.5–98.2)	90.8% (57.2–98.2)	98.8% (97.6–100)	98.0% (97.7–100)	94.1% (79.2–98.4)	0.989 (0.966–0.999)	0.943 (0.728–0.980)

Brier scores (95% CI): Standard logistic regression (MATLAB): 0.0344 (0.0161–0.1117), Mixed-effects logistic regression: 0.0376 (0.0129–0.1417), Bayesian mixed-effects logistic regression: 0.0344 (0.0154–0.1118).

### ROC analysis and model discrimination

The discriminative power of each model was further assessed using ROC curve analysis. All models demonstrated excellent discrimination with AUC values exceeding 0.98. These high AUC values indicate that each modeling approach can effectively discriminate between tumor and normal tissue based on fluorescence intensity metrics, with minimal differences in performance between modeling strategies.

### Precision, recall, and F1 scores

To further assess model performance with respect to clinical utility, we calculated precision (positive predictive value), recall (sensitivity), and F1 scores for each model (Table 2). These metrics are particularly relevant in the context of surgical margin assessment, where both false positives (leading to excessive tissue removal) and false negatives (leading to incomplete tumor resection) have significant clinical consequences. All models demonstrated excellent balance between precision and recall, with F1 scores exceeding 0.94.

### Model calibration

To evaluate how well the predicted probabilities aligned with observed outcomes, we calculated Brier scores for each model. Using the test dataset, the standard logistic regression models (glmfit and LOG, identical models) and BRMS model achieved a Brier score of 0.0344; the GLMER model Brier score was slightly higher (0.0376). All models demonstrated calibration scores substantially below 0.25, indicating well-calibrated predictions. Calibration plots revealed that the standard logistic regression models tended to produce slightly overconfident predictions at the upper and lower extremes of the probability range, while the mixed-effects models, particularly the Bayesian approach, produced more conservative probability estimates that more closely aligned with observed outcomes across the full range of prediction values (Supplementary Fig.2). To assess performance under a sensitivity-constrained condition favoring detection of potential positives, we forced sensitivity to 95% in the test data and evaluated resulting changes in specificity and other metrics (Supplementary Table 2), which increased the accuracy slightly. Performance metrics of the calibrated model for test data with optimal threshold and forced 0.95 sensitivity yielded similar results (Supplementary Table 2).

### Model comparisons for predictive consistency

To compare predictive consistency across modeling frameworks, we randomly selected 16 SBR values - one per patient, including 8 tumor SBRs and 8 normal SBRs to have a balanced dataset and minimize selection bias. Patients 1–8 contributed normal tissue SBR values; patients 9–16 contributed tumor tissue SBR values (Supplementary Table 3). These SBR values were compared. The tumor probability output from these SBRs was calculated and compared across each of the predictive models (standard logistic regression, mixed-effects and Bayesian mixed effects logistic regression) as shown in Supplementary Table 3. Model performance metrics demonstrate the relative effectiveness of different analytical approaches for SBR-based tissue classification, providing insights into optimal modeling strategies for fluorescence-guided tumor detection in clinical applications. All four models consistently assigned high probability scores (≥ 91%) to tumor-associated SBRs, particularly those above SBR 5.0. The mixed effects and Bayesian models accounted for patient-specific variations in baseline fluorescence patterns. For normal tissue, standard logistic regression models produced slightly higher baseline probabilities (0.1–2.3%), while the Bayesian model output remained near zero in most normal cases, indicating conservative calibration. Mixed-effects modeling slightly increased predicted tumor probability in intermediate SBRs (SBR 4 and SBR 8), reflecting the influence of patient-level variability. Minor differences in threshold sensitivity were observed, particularly in intermediate SBRs, where the mixed and Bayesian models produced more conservative predictions.

### Selection of final tumor probability mapping model

Based on a comprehensive evaluation of classification accuracy, discrimination, calibration, and clinical utility metrics, the glmfit model was selected as the optimal approach for developing the final TPM framework.

### The standard logistic regression (MATLAB, glmfit) model is described as

1 $$\:{\boldsymbol{\pi\:}}_{\boldsymbol{i}}=\frac{1}{1\:+\:{\boldsymbol{e}}^{-\left(\:-7.285\:+\:1.617\:\cdot\:\boldsymbol{M}\boldsymbol{e}\boldsymbol{a}\boldsymbol{n}\boldsymbol{S}\boldsymbol{B}{\boldsymbol{R}}_{\boldsymbol{i}}\right)}}$$

The standard logistic regression (MATLAB, glmfit) model (Eq. 1) captures the strong positive relationship between MeanSBR and tumor probability. In this model, $\:{\pi\:}_{i}$ is the predicted probability that the i^th^ region (new ROI/SBR) is a tumor; $\:{\beta\:}_{0}$ (Intercept) = -7.285; β_1_ (slope) = 1.617; and MeanSBR_i,_ is the mean signal-to-background ratio for that tissue region’s MFI divided by the MFI of the background tissue ROI from the same patient. The probability outputs from these models form the foundation of the TPM approach, providing surgeons with quantitative, spatially resolved information regarding the likelihood of tumor presence at each assessed location.

### Demonstrating tumor probility maps developed from glmfit model overlaid onto ex-vivo specimens

**Fig. 4:**
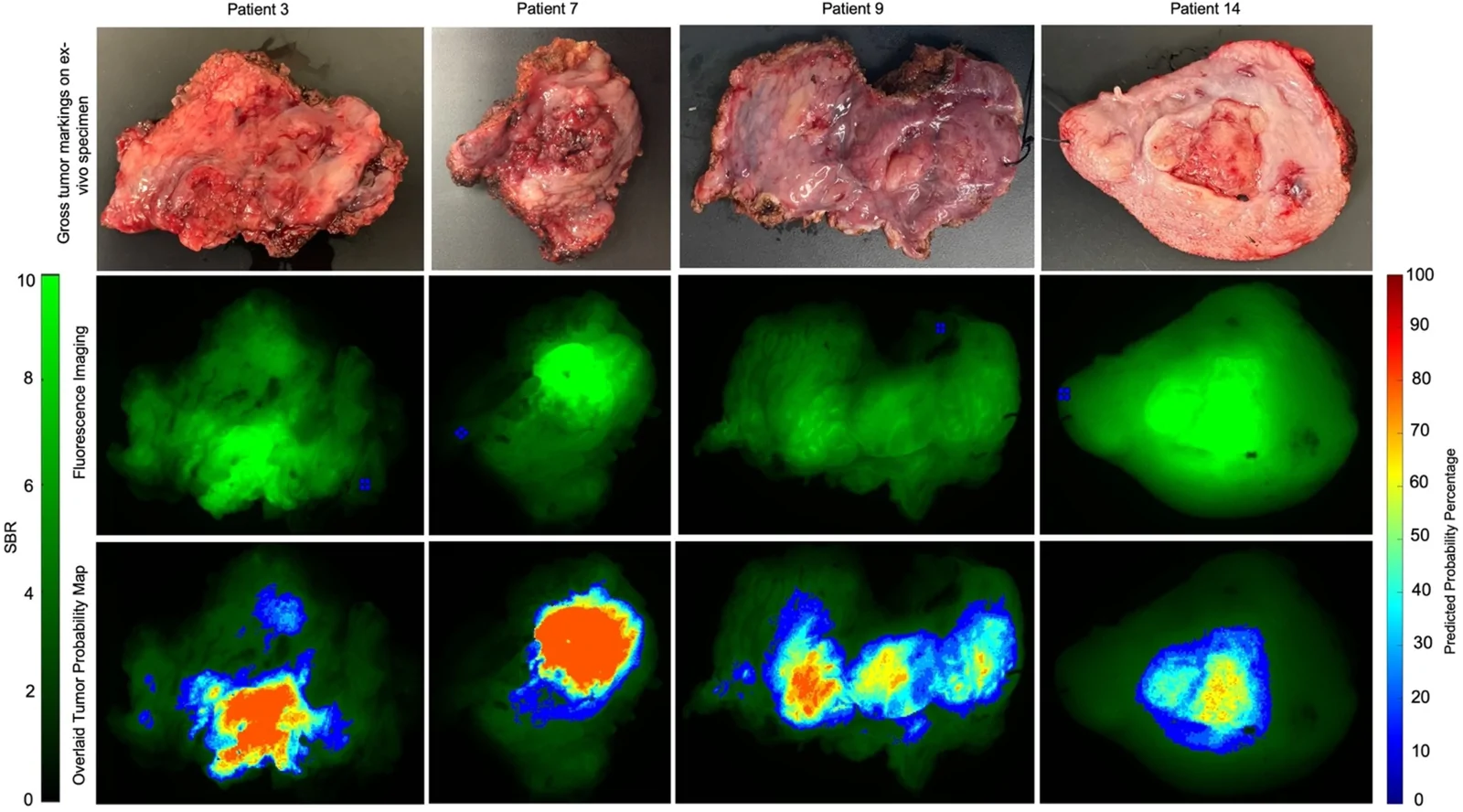
Tumor probability mapping overlaid on ex-vivo fluorescence images of resected tissues. Representative tumor probability maps using SBR-based standard logistic regression (MATLAB) model on resected tumor tissues from four head and neck cancer patients to show the range of TPM performance patterns across the full cohort (Patient 3, 7, 9, and 14). **Upper panels** show gross tumor images of the ex-vivo specimens. **Middle panels** show fluorescence imaging of tissue specimens for each patient with the areas identified as the background (marked by blue squares) which are confirmed as non-tumor tissue based on histopathology. **Lower panels** display corresponding SBR-based predicted tumor probability maps ranging from low probability (darker blue regions) to high probability (brighter yellow/red regions) overlaid on the tissue fluorescence images.

Fluorescence images of resected tumors from four representative patients demonstrate the importance of choosing an accurate background in generating tumor probability maps (Fig. 4). As shown here, the automated TPM approach enabled visualization of calculated probabilities for tumor distribution regions within heterogeneous tissue specimens. The four patients shown illustrate the range of TPM performance patterns observed across the full cohort, specifically: cases where TPM is concordant with clinical and pathological assessment (Patients 3 and 9); a case where TPM over-represents the tumor extent (Patient 7); and a case where TPM under-represents the tumor extent (Patient 14).

**Fig. 5:**
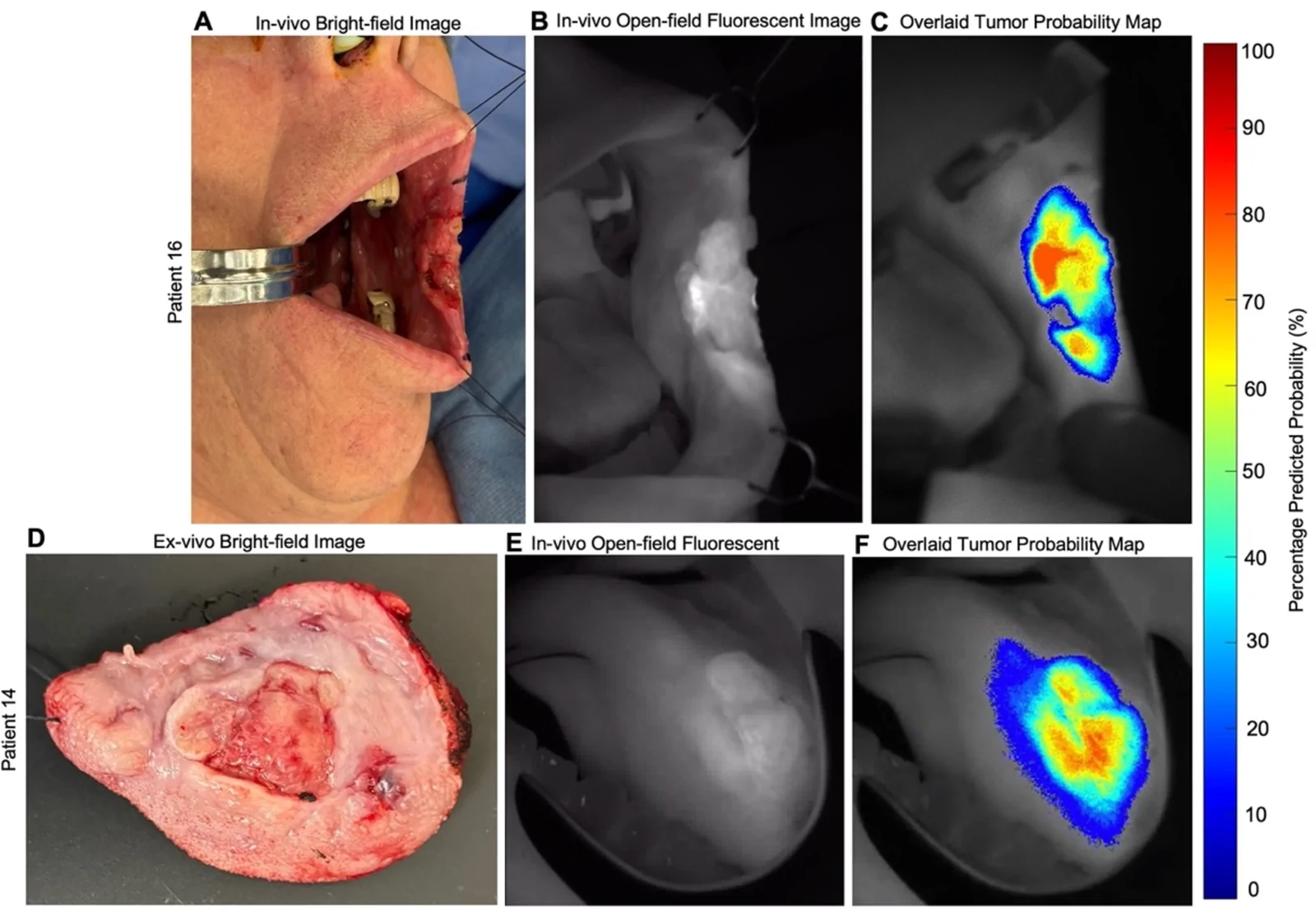
Proof-of-concept tumor probability mapping overlaid on intraoperative open-field fluorescence images. Representative tumor probability maps using SBR-based standard logistic regression (MATLAB) model in two head and neck cancer patients (patients 16 and 14). **A**,** D.** Intraoperative bright-field images. **B**,** E.** Real time fluorescence imaging intraoperatively. **C**,** F.** SBR-based tumor probability maps ranging from low probability (darker blue regions) to high probability (brighter yellow/red regions) overlaid on the tissue fluorescence images.

We also explored the application of TPM for intraoperative fluorescence imaging as demonstrated in Fig. 5 and the Supplementary videos 2 and 3. From intraoperative images of patient 16 (upper panels), the TPM was consistent with the gross clinical assessment while the TPM generated from intraoperative images of patient 14 (lower panels) under-predicted the tumor area, similar to that seen in ex-vivo images for this specific patient (Fig. 4). The histopathology-confirmed tumor tissue SBR values of patient 14 ranged from 4.43 to 9.36. The lower valued tumor SBRs were assigned a low percentage probability of being tumor by logistic regression, giving an underrepresented TPM. Though areas assigned 100% TPM probability over the intraoperative images and videos were consistent with areas clinically assessed as malignant, TPM assigned lower percentage probability of being tumor within additional diseased tissue regions required for resection. Overall, these results demonstrate feasibility of TPM using SBR-based fluorescence analysis for real-time tissue characterization during surgical procedures.

## Discussion

This study conceptualizes TPM as a refined implementation of SBR to enhance intraoperative tumor delineation in HNC surgery. By correlating fluorescence intensity with histopathological validation, we have developed a quantitative model capable of providing surgeons with probability information regarding tumor presence during surgery, to aid in assessment of resection margins. Our findings show that panitumumab-IRDye800-based fluorescence imaging, when analyzed through standardized SBR metrics and integrated into predictive models, offers a reliable method for discriminating tumor from normal tissue during surgical procedures. This approach builds upon previous works^24 and (25^, which demonstrated the utility of fluorescence intensity quantification in cancer margin delineation. We present here a framework for developing histopathology-validated SBR values that can be used to map the tumor in real-time using fluorescence imaging, irrespective of the fluorescence imaging agent, tumor type, or imaging procedure. The logistic regression formula for TPM prediction will produce unique intercept and slope values as imaging parameters are varied, but the overall model and analytical framework are hypothesized to remain the same across varied fluorescence imaging applications, which is a novel aspect of the TPM approach. The methodology described in this study can be applied to other cancer types like ovarian, lung and brain malignancies where fluorescence guidance is routinely being incorporated in surgical management.

The TPM output is a logistic regression-derived estimate that a given tissue region, based on its observed SBR, contains tumor. A probability of > 0.90 indicates that SBR is firmly within the distribution of confirmed tumor ROIs in our training cohort, while a probability of < 0.10 places the region confidently within the normal tissue distribution. The clinically challenging zone is the intermediate probability range (0.20–0.60), which corresponds to SBR values of roughly 3 to 5 in our dataset. Guided by the surgeon’s judgment, these regions with moderate TPM values would warrant additional evaluation such as FSA to confirm the presence or absence of tumor cells.

The achievement of negative surgical margins remains one of the most significant modifiable prognostic factors in HNC surgery, with significant associations between positive margins and decreased survival outcomes^2,6,26–32^. Our study extends beyond binary threshold approaches by implementing multiple logistic regression models for tumor prediction. The TPM approach addresses critical limitations of current margin assessment techniques by providing a percentage probability rather than a binary threshold for tumor presence. This method helps in assisting the surgeon to independently make this decision using fluorescence as guidance. Critically, TPM is designed as a decision support tool, not a replacement for surgeon judgment or pathological assessment. This is consistent with FDA guidance for surgical visualization devices, which require that such tools enhance rather than replace the surgeon’s own assessment^33^. By converting fluorescence intensity data into a clinically meaningful probability map, TPM bridges the gap between qualitative fluorescence imaging and actionable surgical guidance^34–37^. The identification of 5,442 ROIs across tissues from 16 patients allowed for robust model training and testing, demonstrating 98% specificity. The analysis demonstrated that SBR values strongly correlate with tumor presence when validated against the gold standard histopathology. The present proof-of-concept study was developed with historical data where we have true histopathology to choose a background from non-tumor tissue areas. Our hypothesis is that in real-time settings, tissue margins confirmed as negative for tumor during frozen section analysis can be considered as background for calculating MeanSBR to generate TPM images. This correlation provides the foundation for a reliable probability model that can be applied intraoperatively to guide surgical decision-making without the time/procedural delays, discordance rates and sampling errors associated with FSA.

Unlike FSA, which examines discrete (piece-meal) tissue samples, TPM offers comprehensive assessment across the entire surgical field. Our fluorescence results of resected tissues are consistent with the meta-analysis by Higginson et al.^17^, which demonstrated that tumor-targeted fluorescence imaging has excellent pooled sensitivity and specificity of 95.7% and 82.7%, respectively, for tumor detection. Logistic regression of fluorescence SBR values in the present study achieved 90.8% sensitivity and 98.8% specificity on test data (Table 2), which compares favorably. Other IMA techniques reported in the meta-analysis by Higginson et al. yielded respective pooled sensitivities and specificities of 79.5% and 99.1% for FSA, 91.9% and 85.5% for optical techniques (narrow band imaging, optical coherence tomography, etc.), 92.5% and 98.8% for touch imprint cytology, and 91.8% and 75.9% for topical staining^17^. However, we caution that direct numeric comparisons across studies must be interpreted carefully given differences in patient selection, tumor type, imaging systems, and the definition of a positive malignant result (margin, tumor region, etc.). Recent works further support the clinical value of FGS in reducing positive margin rates in HNC resections^19,38^. Future prospective trials would be required to evaluate accuracy and clinical impacts of TPM, alone or in combination with other IMA approaches, for detecting positive margins during surgery.

Current fluorescence imaging techniques often rely on subjective interpretation of signal intensity^39^. A technical innovation of TPM includes its ability to normalize fluorescence signals against optical properties of background tissue, thereby accounting for inter-patient variability and tissue heterogeneity. This methodology aligns with recommendations by Koch, Ntziachristos and Vahrmeijer et al.^38,40^, who advocated for standardized quantification methods in FGS to overcome the limitations of subjective visual assessment. The integration of fluorescence data with predictive modeling represents a significant advancement over current binary threshold-based approaches. Rather than designating tissue as either tumor or normal based on a single cut-off value, TPM provides a continuous probability that can be tuned to clinical context. A surgeon prioritizing sensitivity (reducing false negatives) may decide to act on any region with probability > 0.20, accepting more re-excision. Whereas, a surgeon in a functionally critical location (e.g., base of tongue adjacent to the hypoglossal nerve) may prefer a higher probability threshold. Therefore, any intermediate-probability region flagged by TPM should be treated as a candidate for additional sampling, just as a visually ambiguous region would be. This gradient information is particularly valuable in areas where tumor cells intermingle with normal tissue, creating a transition zone that is challenging to delineate with conventional techniques. Given that IMA modalities possess complementary strengths in evaluating tumor boundaries, future studies should prospectively evaluate TPM using FI within a multimodal IMA framework to determine whether integrating these approaches improve detection of small satellite tumor islands, metastatic deposits, and second primary tumors that may be missed by routine visual inspection.

The concept of TPM generation has also been explored in the context of deep learning-based segmentation, as demonstrated by De Biase et al., who developed a graphical user interface for visualizing probability maps in head and neck cancer imaging to enhance interpretability and clinical integration^41^. Similarly in this study we evaluated MeanSBR fluorescence-based TPM with four predictive modeling approaches and compared them for performances. While the standard logistic regression model had high AUC values, the mixed effects logistic regression model accounted for intra-subject correlation, improving robustness in the presence of repeated measures. The Bayesian model provided additional interpretability through posterior distributions, making it suitable for settings where uncertainty quantification is critical. Overall, the standard logistic regression model offered a simple and efficient method for real-time intraoperative application, whereas the hierarchical and Bayesian approaches may be more appropriate in research or exploratory settings where modeling complexity and interpretability are priorities. This comparative analysis highlights consistent tumor probability mapping performance across models and illustrates their calibration tendencies, aligning with prior studies that emphasize the value of evaluating diverse modeling frameworks to enhance intraoperative fluorescence-guided diagnostics and support clinical decision-making^42,43^. Overall, the outputs showed strong alignment across models for highly discriminative SBRs while revealing subtle differences in threshold sensitivity and probabilistic spread, particularly in borderline or moderate-intensity regions.

Despite the promising results, several limitations should be acknowledged. First, the current study was limited to 16 patients, which may not fully capture the heterogeneity of HNC. Further validation with a larger cohort from multiple centers will be necessary to establish the generalizability of our findings across different tumor subtypes, stages, and patient demographics. Second, while panitumumab-IRDye800 targets EGFR, overexpressed in many HNCs, not all tumors demonstrate uniform EGFR expression^44^. Variability in receptor density, expression and binding potential may affect the reliability of fluorescence signals in certain tumor regions. Future iterations of TPM may benefit from incorporating multiple targeted fluorophores to address this heterogeneity, an approach supported to improve tumor detection using a combination of targeting strategies^45^. In addition to variable receptor expression on cancer cells, potential reasons for artificially low probability assignments associated with the FI-based TPM approach include tissue regions with low tumor cell density, tumor regions with dense stromal features, low vascularity, hypoxia or necrosis^46^. Overestimation of tumor probability can occur in peri-tumoral tissue that has high native EGFR expression, resulting in SBR values approaching those in malignant regions. Third, the standardization of imaging parameters and ROI selection requires careful attention to methodology. Differences in tissue thickness, imaging timing relative to contrast agent administration, and imaging system settings can all influence fluorescence intensity measurements. We have attempted to control for these variables through rigorous standardization, but implementation in diverse clinical settings will require robust protocols to ensure consistency. This study did not assess edge cases such as satellite tumor foci; model performance specific to the tumor margins was also not assessed and is worthy of evaluation in future work. Additionally, this proof-of-concept study was performed after surgical and pathology procedures were completed; future work is needed to implement the fluorescence-based TPM approach during surgery in real time. Finally, while our approach provides probability information, the ultimate determination of resection margins remains a clinical decision. TPM should be viewed as a complementary tool to enhance surgical visualization rather than a replacement for surgical expertise or pathological assessment.

The development of TPM offers promising clinical applications, including integration with surgical navigation systems to provide real-time tumor probability maps and enhance intraoperative decision-making. It could also streamline pathological grossing by identifying tumor-rich sections, improve margin assessment, and aid in lymph node evaluation and sentinel node identification. Recent advances in augmented reality surgical navigation systems^47^ further highlight the feasibility of such integrated approaches. Expansion of predictive models to incorporate additional patient-specific factors such as tumor grade, EGFR expression levels, and previous treatment history could further refine probability assessments. A recent study by Nijboer et al. focuses on clinical feasibility and surgical outcome improvement using fluorescence-guided FSA for margin analyses^48^, while our proof-of-concept study emphasizes probabilistic modeling and uncertainty quantification of spatial TPM derived from fluorescence SBR. The two approaches provide different but synergistic information to clinicians during FGS. Machine learning approaches that combine fluorescence data with clinical variables may improve the accuracy of tumor prediction and enable personalized threshold recommendations based on individual risk profiles. TPM could be incorporated at multiple stages of fluorescence-guided HNC surgeries due to the lifetime property of the fluorescence. TPM could also potentially be used during imaging of the surgical wound bed post resection to elucidate satellite/micro-metastasis, imaging of the frozen section and gross specimens to guide pathologists in accurate sampling, and further in fluorescence microscopy of histopathological tissue sections to complement H&E analysis.

Longitudinal studies correlating intraoperative TPM findings with long-term outcomes such as local recurrence rates and overall survival will be essential to establish the clinical impact of this approach. Such studies would provide the evidence base necessary for broader adoption of FGS with TPM as a standard of care in HNC management. The ongoing multi-institutional trials provide a methodological framework for such validation studies, which will be crucial for clinical translation^16^. Technical refinements in imaging systems, including higher spatial resolution and improved sensitivity, will continue to enhance the capabilities of TPM. Development of dedicated software tools for automated ROI analysis and real-time probability calculation would facilitate clinical implementation by reducing the computational burden on surgical teams.

TPM approach developed here represents a significant advancement in the field of FGS for HNC. By transforming qualitative fluorescence signals into quantitative probability assessments validated against histopathology, TPM provides surgeons with valuable information to guide resection decisions while remaining within FDA guidelines for surgical visualization tools. This approach addresses critical limitations of current margin assessment techniques and has the potential to improve surgical outcomes by reducing positive margin rates and identifying occult tumor extensions. While further validation and technical refinement are needed, TPM demonstrates promising utility as a complement to existing surgical and pathological methods in the pursuit of complete tumor resection with clear margins, ultimately addressing the clinical challenge of margin control.

## Supplementary Information

Below is the link to the electronic supplementary material.

supplementary material 1

supplementary material 2

supplementary material 3

## Data Availability

The data that support the findings of this study are available on request from the corresponding author.

## Abbreviations

FI: Fluorescence imaging
TTF: Tumor-targeted fluorescence
FGS: Fluorescence-Guided Surgery
HNC: Head and Neck Cancer
MFI: Mean Fluorescence Intensity
SBR: Signal-to-Background Ratio
LRR: Local recurrence rate
DFS: Disease-free survival
TPM: Tumor probability mapping
FSA: Frozen section analysis
IMA: Intraoperative margin assessment
