# Supplementary material for: Tumor probability mapping of fluorescence intensity during image-guided surgery of head and neck cancer
Source: Sci Rep. 2026 Jun 5;16:28969. doi: 10.1038/s41598-026-56078-4 (PMC13582841; doi:10.1038/s41598-026-56078-4)
Supplement: Supplementary file 1 [file 41598_2026_56078_MOESM1_ESM.pdf]

Supplementary Data:

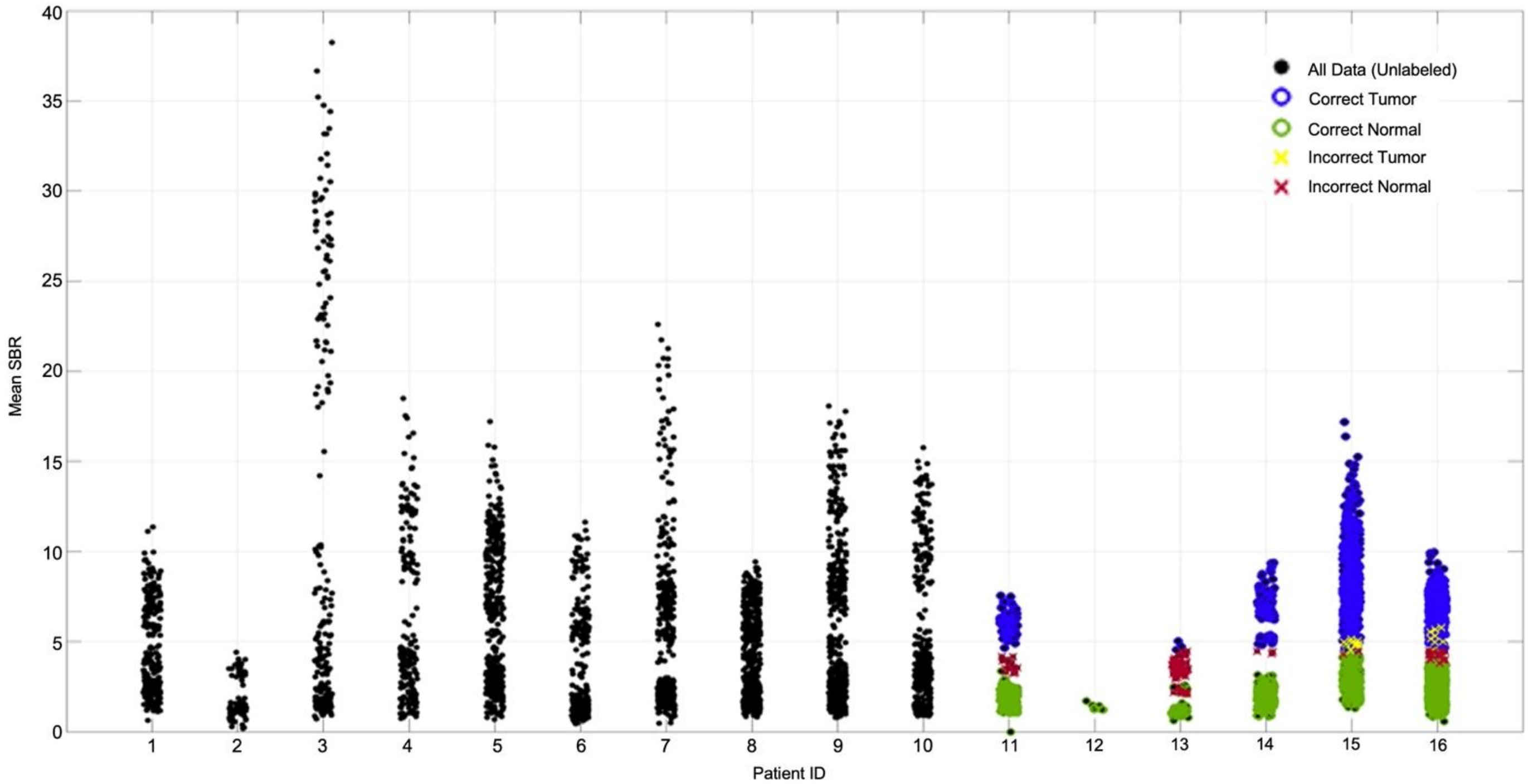

Supplementary Figure 1. Detailed classification performance analysis of SBR-based tumor prediction model. Comprehensive dot plot displaying classification results for all SBR measurements from 16 head and neck cancer patients, illustrating model performance through color-coded prediction outcomes. **Black dots** represent the complete dataset of SBR values from both tumor and normal tissue measurements across all patients. Classification results are color-coded based on prediction accuracy: **blue dots** indicate true positives (correctly predicted tumor tissue), **green dots** represent true negatives (correctly predicted normal tissue), **yellow X's** show false positives (normal tissue incorrectly classified as tumor), and **red X's** indicate false negatives (tumor tissue incorrectly classified as normal).

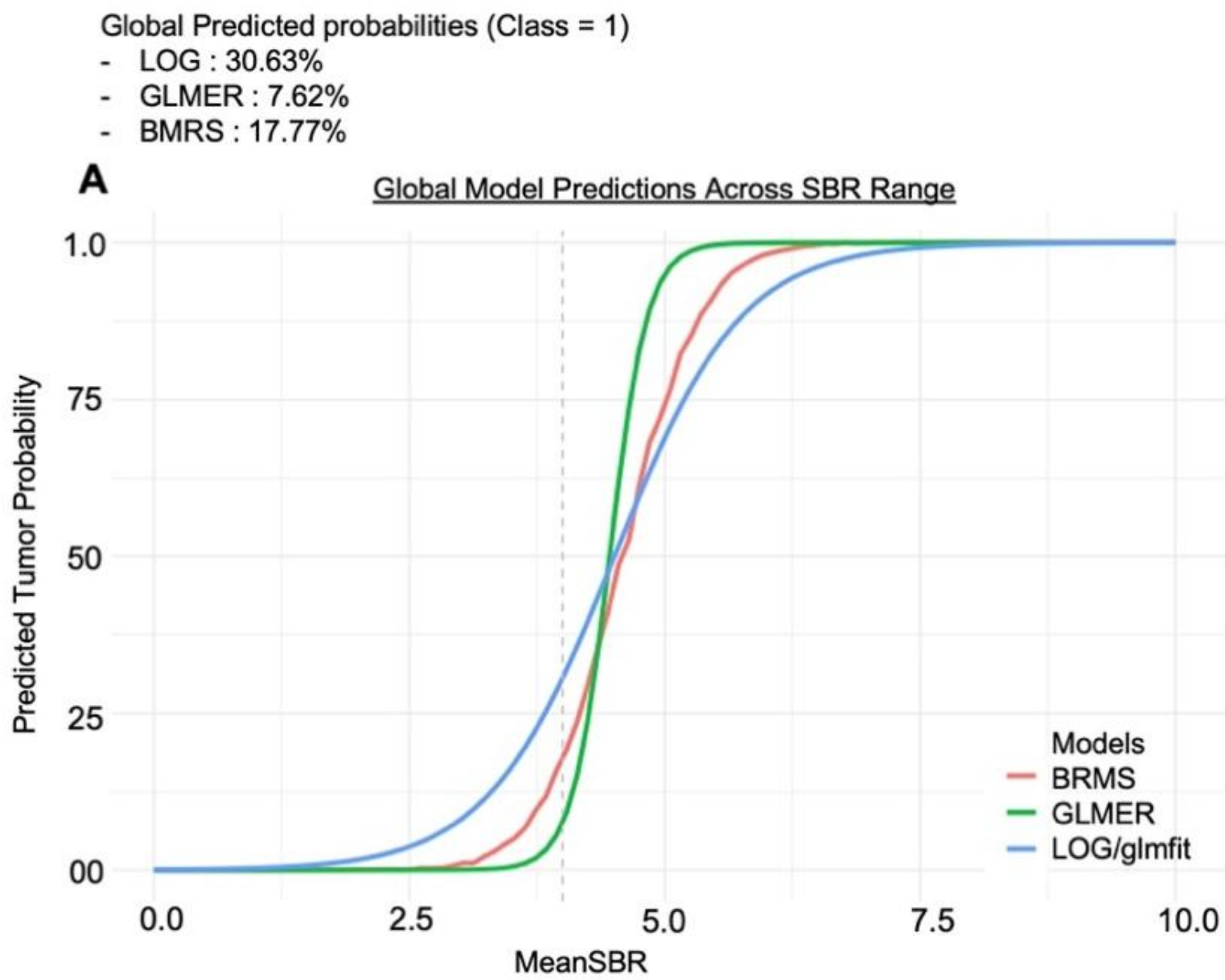

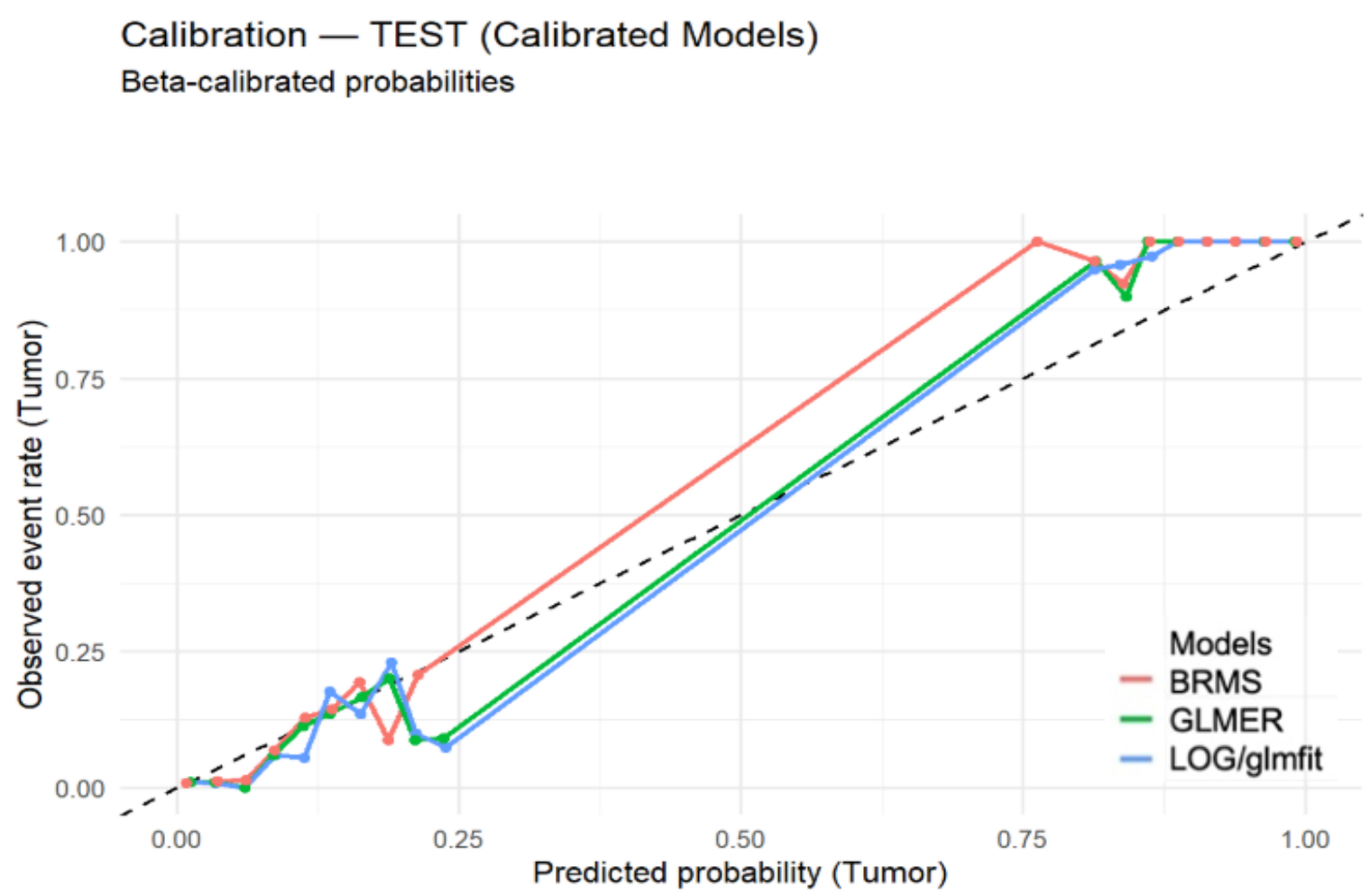

Delong test

```
## # A tibble: 3 × 5
##   Comparison         AUC_1 AUC_2 p_value p_value_adj
##   <chr>              <dbl> <dbl>   <dbl>       <dbl>
## 1 Logistic vs GLMER  0.989 0.989       1           1
## 2 Logistic vs BRMS   0.989 0.989       1           1
## 3 GLMER vs BRMS      0.989 0.989       1           1
```

Supplementary Figure 2. Global model predictions across SBR range and calibration plot. Comparison of model predictions and calibration for tumor probability estimation based on MeanSBR. **(A)** Illustrates predicted tumor probabilities across the MeanSBR range (0–10) for three models: Bayesian mixed-effects model (BRMS - red), Mixed-effects model (GLMER - green), Standard logistic regression model (LOG/glmfit - blue). The y-axis represents predicted tumor probability (0–1). **(B)** Shows calibration plots comparing observed rates to mean predicted probabilities for Bayesian mexed-effects model (BRMS - red), Mixed-effects model (GLMER - green), and standard logistic regression (LOG/glmfit - blue). Points represent grouped observations, and the dashed diagonal line (black) indicates perfect calibration. Global predicted probabilities for class = 1 were 30.63% Standard logistic regression (LOG/glmfit), 7.76% Mixed-effects logistic regression (GLMER), and 17.77% Bayesian mixed-effects logistic regression (BRMS).

Supplementary Table 1. False negative and false positive outcomes per patient in the test set with the MeanSBR and predicted probability % ranges.

Patient ID	Predicted class	True class	Predicted probability % range	MeanSBR range	Total number
False Negative					
11	Normal	Tumor	11.39 - 36.57	3.23 - 4.16	13
13	Normal	Tumor	2.00 - 48.39	2.09 - 4.46	54
14	Normal	Tumor	43.06 - 47.98	4.33 - 4.45	3
15	Normal	Tumor	42.33 - 48.37	4.31 - 4.46	2
16	Normal	Tumor	24.03 - 49.25	3.79 - 4.48	18
					**90**
False Positive					
15	Tumor	Normal	50.98 - 73.42	4.52 - 5.13	9
16	Tumor	Normal	50.81 - 86.66	4.52 - 5.77	9
					**18**

Supplementary Table 2. Performance metrics (95% CI) on test dataset @ calibrated and forced 0.95 sensitivity and @ calibrated and optimal threshold.

Model	Accuracy	Sensitivity (Recall)	Specificity	PPV (Precision)	NPV	AUC	F1 Score
Standard logistic regression (MATLAB)	94.6% (89.0–97.6)	95.8% (72.4–100)	93.8% (87.1–100)	91.3% (89.3–100)	97.1% (86.6–100)	0.989 (0.966–0.999)	0.935 (0.840–0.961)
Standard logistic regression (R)	94.6% (89.0–97.6)	95.8% (72.4–100)	93.8% (87.1–100)	91.3% (89.3–100)	97.1% (86.6–100)	0.989 (0.966–0.999)	0.935 (0.840–0.961)
Mixed-effects logistic regression	94.8% (89.0–97.8)	95.5% (73.8–100)	94.1% (88.2–100)	91.7% (89.7–100)	97.0% (85.5–100)	0.989 (0.966–0.999)	0.936 (0.856–0.964)
Bayesian mixed-effects logistic regression	94.6% (88.8–97.6)	95.8% (74.4–100)	93.8% (87.8–100)	91.3% (89.3–100)	97.1% (86.0–100)	0.989 (0.966–0.999)	0.935 (0.853–0.962)

Brier scores (95% CI): Standard logisitic regression (MATLAB): 0.0344 (0.0161–0.1063), Standard logistic regression (R): 0.0344 (0.0161–0.1059), Mixed-effects logistic regression: 0.0358 (0.0152–0.1094), Bayesian mixed-effects logistic regression: 0.0344 (0.0161–0.1060)

Supplementary Table 3: Comparative performance of four predictive models using SBR values. Model comparison analysis using randomly selected SBR values from 16 head and neck cancer patients to evaluate predictive performance across different analytical approaches. Black font indicates normal tissue ROI, red font indicates tumor tissue ROI.

Tumor percentage probability					
SBR		glmfit MATLAB	Logistic regression	Mixed model	Bayesian model
Coding Software		MATLAB	R		
1	0.184354	0.1%	0.1%	0.0%	0.0%
2	1.869965	1.4%	1.4%	0.0%	0.0%
3	0.980586	0.3%	0.3%	0.0%	0.0%
4	2.02463	1.8%	1.8%	6.4%	6.8%
5	0.949777	0.3%	0.3%	0.0%	0.0%
6	1.66474	1.0%	1.0%	0.0%	0.6%
7	1.309365	0.6%	0.6%	0.0%	0.1%
8	2.195402	2.3%	2.3%	0.0%	5.1%
9	10.22248	100%	100%	100%	100%
10	8.792079	99.9%	99.9%	100%	100%
11	5.970681	91.4%	91.4%	99.9%	99.7%
12	5.821268	89.4%	89.4%	100%	100%
13	7.081081	98.5%	98.5%	100%	99.9%
14	7.92117	99.6%	99.6%	99.2%	97.2%
15	2.137541	2.1%	2.1%	0.0%	4.5%
16	7.571454	99.3%	99.3%	100%	96.0%

Supplemental table 4: Software validation anaylysis (Standard logistic regression - R)

Performance metrics (95% CI) on training dataset @ 0.5 threshold*:*

Model	*Accuracy*	*Sensitivity (Recall)*	*Specificity*	*PPV (Precision)*	*NPV*	*AUC*	*F1 Score*
Standard logistic regression (R)	92.9% (89.4–95.8)	90.5% (82.9–97.2)	94.9% (90.9–98.8)	93.8% (87.7–98.6)	92.2% (85.8–97.8)	0.984 (0.971–0.994)	0.921 (0.877–0.956)

Brier scores (95% CI): 0.0504 (0.0315–0.0735)

Performance metrics (95% CI) on test dataset @ *.5 threshold*

Model	*Accuracy*	*Sensitivity (Recall)*	*Specificity*	*PPV (Precision)*	*NPV*	*AUC*	*F1 Score*
Standard logistic regression (R)	95.5% (84.5–98.2)	90.8% (57.2–98.2)	98.8% (97.6–100)	98.0% (97.7–100)	94.1% (79.2–98.4)	0.989 (0.966–0.999)	0.943 (0.728–0.980)

Brier scores (95% CI): 0.0344 (0.0154–0.1117),

Supplementary Videos #1 and #2:

Power Point presentation with video hyperlinks
